# Integrating Study Designs on Emotional Reactivity and Regulation in Old Age: New Evidence From the EMIL Study

**DOI:** 10.1093/geroni/igab046.1278

**Published:** 2021-12-17

**Authors:** Oliver Schilling, Gloria Luong

## Abstract

Key insights into emotional reactivity and regulation have been gained by studying how these dynamics evolve as older people are confronted with controlled stressors in the lab, go about their everyday routines, or develop across adulthood and old age. Yet, we are only beginning to understand how the dynamics on the different time scales observed in these study designs interact . Aiming for a comprehensive picture of the predictors, correlates, and consequences of emotional reactivity and regulation, the EMIL study integrates a lab-based study with ambulatory in-vivo assessments and a classic long-term longitudinal study. 130 young-old (65-69 years) and 59 very-old adults (83-89 years) from the ILSE study, contributing four waves of health, cognitive, and psycho-social data over almost 25 years, were tested in the lab and assessed six times a day over seven consecutive days. We provide an overview of and first across-design results from EMIL: Katzorreck et al. examined whether the frequency of exposure to daily stressors affects emotion regulation capacity as tested in the lab. Lücke et al. analyzed daily working memory performance, sleep, and its association with long-term change in cognitive functioning. Wieck et al. present differential effects of discrete negative emotions as induced in the lab and reported in daily life on social cognitive performance as indicated by empathic accuracy. Gerstorf et al. examined how long-term cognitive aging affects positive feelings and stressor reactivity in daily life. Gloria Luong will discuss the presentations, considering challenges and opportunities of integrating lab-based, ambulatory, and longitudinal study designs.

